# Enhancing Oral Health Knowledge Among Elementary School Teachers: Outcomes of A Quasi‐Experimental Educational Intervention

**DOI:** 10.1111/josh.70062

**Published:** 2025-08-08

**Authors:** Taís Zacaria, Fernanda Ruffo Ortiz

**Affiliations:** ^1^ ATITUS‐Educação Passo Fundo Brazil

**Keywords:** educational intervention, elementary school teachers, oral health knowledge, outcomes

## Abstract

**Background:**

Knowledge about oral health becomes essential, especially during school years when the prevalence of dental problems is high. The objective was to assess whether an educational intervention improves teachers' knowledge of oral health.

**Methods:**

The sample was obtained through a census of elementary school teachers from public schools in Brazil. A structured questionnaire, consisting of questions about demographic data, knowledge of dental caries and periodontal disease, oral hygiene practices, preventive dentistry, and attitudes toward oral health, was administered three times: before, immediately after, and three months after the educational intervention. An educational intervention was conducted through a face‐to‐face session (1‐h lecture) in all schools, addressing oral health topics (aforementioned). Descriptive analysis and comparison tests (McNemar and Wilcoxon) were employed to assess the impact of the intervention on teachers' knowledge.

**Results:**

Eighty‐three teachers agreed to participate (89% adherence rate), and 53 teachers completed all three questionnaires (64% final retention rate). The results showed that the intervention improved teachers' knowledge immediately after and three months after the intervention (*p* < 0.05) in responses related to knowledge of dental caries, periodontal disease, oral hygiene practices, and preventive dentistry.

**Conclusions:**

This study demonstrated that teachers' knowledge of oral health improved after the intervention.

## 1 Introduction

Oral health is an essential component of general health and a critical factor in an individual's quality of life [1]. Conditions such as dental caries, periodontal disease, malocclusion, and dental trauma can significantly affect children's quality of life [2], causing pain, discomfort, and potentially leading to the loss of teeth [3]. In many municipalities in Brazil, especially in public schools, students show high rates of dental caries and poor oral health [4].

During childhood, a period when habits are formed, children spend a substantial amount of time in school. Primary schools play a key role in delivering oral health information, as they can directly influence children's health behaviors. In this context, teachers assume a vital role in these stages of child development [5]. Therefore, schools serve as ideal settings for implementing oral health education programs, promoting consistent and inclusive access for all children [6, 7].

Educational and preventive programs targeting elementary school teachers in different regions have demonstrated significant improvements in teachers' oral health knowledge and practices [8, 9, 10, 11, 12, 13]; although these improvements are often short‐term [14]. Two studies conducted in India reported a significant increase in oral health knowledge after a face‐to‐face educational program [11] and improved knowledge about the effect of sugars on oral health in an online format [8]. These studies also show that even simple interventions can benefit healthy behaviors [15]. However, according to previous literature, there have been few intervention studies conducted with teachers [8, 11]; and no studies have been conducted in Brazil.

Given their potential to influence children's health behaviors, teachers may play an important role in health promotion. Providing them with training in oral health, supported by active participation from dentists, could enhance the effectiveness of such initiatives. Therefore, this study aims to evaluate whether an educational intervention improves elementary school teachers' knowledge of oral health. Our conceptual hypothesis is that teachers' knowledge of oral health will improve after the educational intervention.

## 2 Methods

A quasi‐experimental intervention study was conducted in 2024 with elementary school teachers who taught students from grades 1 to 5. This age group was selected because it is a critical period when personal habits and characteristics are formed; children at this stage are more receptive to new information and tend to absorb and incorporate it more easily [5].

This study was submitted and approved by the Research Ethics Committee of Atitus Educação (CAAE 75352723.0.0000.5319).

The research was carried out in Veranópolis, a city in the Serra Gaúcha region of Rio Grande do Sul, Brazil, which had a population of 24,021 in 2022 [16]. At the time of data collection, the city had seven elementary schools with a total of 93 teachers. All schools meeting the eligibility criteria (municipal public elementary schools serving grades 1 to 5) were invited and agreed to participate. Teachers in training or interns were excluded from the study. A total of 93 eligible teachers were invited to participate in the study and provided their consent.

Data collection was through a structured, self‐administered online questionnaire on oral health, distributed at three separate times. Initially, teachers received the questionnaire in Brazilian Portuguese via Google Forms and responded based on their existing knowledge without prior information from the researchers. Afterward, the teachers received the educational intervention, and the same questionnaire was sent to assess changes in knowledge immediately following the intervention and again three months later to evaluate knowledge retention. The content of the questionnaire remained consistent across all three administrations. According to previous literature, the time between the pre‐test, intervention, and post‐test in health interventions is four to eight weeks [17]. Therefore, the three‐month follow‐up was designed to assess longer‐term retention.

The questionnaire was based on previous studies [13, 18, 19, 20, 21, 22] consisted of 25 multiple‐choice questions covering: (1) demographic data (age, gender identity, education level, and years of teaching experience); (2) knowledge about caries and periodontal disease (definition, prevention, related foods, biofilm, and gingival bleeding); (3) oral hygiene practices and preventive dentistry (information sources, tooth eruption, brushing frequency and technique, use of fluoride, and dental visits); and (4) attitudes toward oral health and education (importance of treating primary teeth, oral‐systemic health relationship, and promotion of oral health in schools). In the post‐test, two additional questions assessed perceived knowledge gain and satisfaction with the training.

The educational intervention was delivered face‐to‐face in a single, approximately one‐hour session at each of the seven schools during teachers' break times. The presentation, supported by slides, covered topics such as: dental biofilm (what it is, how it forms, and removal techniques), dental caries (what it is, prevention, and diet's influence), periodontal diseases, oral hygiene in pediatric dentistry (tooth brushing, flossing, toothpaste use, when to take a child to the dentist), and oral health education (activities to promote oral health knowledge through playful activities) [21, 23].

A descriptive analysis of the teacher's responses was performed. Qualitative variables were expressed in frequencies and percentages, and quantitative variables were measured in central tendency and dispersion measures (standard deviation—SD). A comparison test was performed to assess responses before and after the educational intervention using McNemar's test (nominal qualitative variables) and the Wilcoxon test (ordinal qualitative variables) for dependent samples. The results were interpreted using a significance level of 5%. Data analyses were performed using STATA statistical software version 14.

During the revision process of this work, the authors used ChatGPT (OpenAI) to check the spelling, grammar, and fluency of the text. This tool was not used to generate content. Following the tool's suggestions, the authors revised and edited the content, taking full responsibility for the content of the article.

## 3 Results

A total of 93 teachers were invited to participate in the study. Of these, 83 agreed and completed the pre‐test (89% participation rate). After the intervention, 79 teachers responded to the immediate post‐test (95% retention rate), and 53 teachers completed all three questionnaires (pre‐test, immediate post‐test, and three‐month post‐test) (64% final retention rate).

All the participants (100%, *n* = 83) were female, with an average teaching experience of 18.5 years (SD = 8.22). The level of education of the teachers was teacher training (11.9%), undergraduate (46.43%), specialization (50.0%), master's (11.9%), and post‐doctorate (11.9%) (data not shown in the table).

Table 1 compares the responses from the pre‐test, immediate post‐test, and three‐month post‐test. Initially, teachers showed insufficient knowledge about oral health in the pre‐test, particularly in questions regarding caries prevention, dental biofilm, consequences of biofilm accumulation, appropriate toothpaste amount, and the necessity of treating caries in primary teeth.

**TABLE 1:** Responses obtained in pre‐tests, immediate post‐tests and post‐tests (3 months later).

	Pre‐test	Immediate Post‐test	*p*‐value^a^	Post‐test 3 months	*p*‐value^c^
	*n* (%)	*n* (%)		*n* (%)	
1. What is dental caries?			**0.005**		0.654
Incorrect answer	17 (20.48)	05 (6.33)		03 (5.66)	
Correct answer	66 (79.52)	74 (93.67)		50 (94.34)	
2. How to prevent dental caries?			**< 0.001**		**< 0.001**
Incorrect answer	48 (57.83)	70 (88.61)		06 (11.32)	
Correct answer	35 (42.17)	9 (11.39)		47 (88.68)	
3. Which foods are directly related to the appearance of caries?			0.285		0.179
Incorrect answer	10 (12.05)	5 (6.33)		4 (7.55)	
Correct answer	73 (87.95)	74 (93.67)		49 (92.45)	
4. What is dental biofilm?			0.116		1.000
Incorrect answer	63 (75.90)	68 (86.08)		13 (24.53)	
Correct answer	20 (24.10)	11 (13.92)		40 (75.47)	
5. How can dental biofilm be removed?			**< 0.001**		**< 0.001**
Incorrect answer	41 (49.40)	12 (15.19)		16 (30.19)	
Correct answer	42 (50.60)	67 (84.81)		37 (69.81)	
6. What does the accumulation of dental biofilm cause?			**< 0.001**		**0.001**
Incorrect answer	47 (56.63)	9 (11.39)		16 (30.19)	
Correct answer	36 (43.37)	70 (88.61)		37 (69.81)	
7. Bleeding gums or gingivitis means?			**0.008**		—
Incorrect answer	15 (18.07)	3 (3.80)		—	
Correct answer	68 (81.93)	76 (96.20)		53 (100)	
8. What sources did you use to obtain information about oral health?			**< 0.001**		
Magazines	—				
Internet	44 (53.01)				
Dentist	2 (2.41)				
University	—				
Family	—				
Internet + Dentist	21 (25.30)				
Internet + Newspaper	7 (6)				
Internet + Dentist + University	8 (7)				
Newspapers and dentist	—				
Internet and university	3 (3.61)				
9. At what age does the first permanent tooth erupt in children?			0.738		0.738
Incorrect answer	5 (6.02)	4 (5.06)		5 (9.43)	
Correct answer	78 (93.98)	75 (94.94)		48 (90.57)	
10. What is the recommended frequency of brushing for children?			**< 0.001**		**< 0.001**
Incorrect answer	39 (46.99)	6 (7.59)		16 (30.19)	
Correct answer	44 (53.01)	73 (92.41)		37 (69.81)	
11. What is the necessary amount of toothpaste for brushing for children over 3 years old?			**< 0.001**		**< 0.001**
Incorrect answer	47 (56.63)	70 (88.61)		12 (22.64)	
Correct answer	36 (43.37)	9 (11.39)		41 (77.36)	
12. What is most important in brushing?			**0.007**		**0.001**
Incorrect answer	20 (24.10)	5 (6.33)		10 (18.87)	
Correct answer	63 (75.90)	74 (93.67)		43 (81.13)	
13. What is the ideal age to take a child to the dentist for the first time?			0.479		0.179
Incorrect answer	3 (3.61)	5 (6.33)		4 (7.55)	
Correct answer	80 (96.39)	74 (93.67)		49 (92.45)	
14. Do you know the importance of fluoride in oral health?			—		—
Incorrect answer	—	—		—	
Correct answer	83 (100)	79 (100)		53 (100)	
15. What is the function of fluoride?			0.179		0.157
Incorrect answer	1 (1.20)	4 (5.06)		2 (3.77)	
Correct answer	82 (98.80)	75 (94.94)		51 (96.23)	
16. Is oral health related to general health?^b^			—		0.593
Partially agree	9 (10.84)	4 (5.06)		6 (11.32)	
Totally agree	74 (89.16)	75 (94.94)		47 (88.67)	
17. Is it necessary to treat caries in primary teeth (baby teeth)?^b^			**< 0.001**		0.007
Partially agree	46 (55.42)	8 (10.13)		13 (24.53)	
Totally agree	37 (44.58)	71 (89.87)		40 (75.47)	
18. Have you ever conducted any activity related to knowledge of oral health with your students?			0.763		0.317
No	9 (10.84)	7 (8.86)		4 (7.55)	
Yes	74 (89.16)	72 (91.14)		49 (92.45)	
19. Do you think it is important for your students to be educated in school about their oral health?			—		—
No	1 (1.20)	—		—	
Yes	81 (97.59)	79 (100)		—	
I don't want to answer	1 (1.20)	—		—	
20. Do you think it is important to have guidance on educational and preventive measures for promoting your students' oral health?			—		—
No	2 (2.41)	—		—	
Yes	80 (96.39)	79 (100)		—	
I don't want to answer	1 (1.20)	—		—	

^a^ McNemar test.

^b^ Wilcoxon test.

^c^ McNemar or Wilcoxon Test between Pre‐test and Post‐test after 3 months.

*Note*: Values in bold indicate statistical significance.

Significant improvements (*p* < 0.05) were identified between the pre‐test and immediate post‐test in knowledge of dental caries, periodontal disease, oral hygiene practices, preventive dentistry, attitude toward oral health, and oral health education. Thus, it is observed that the participants changed their answers to the correct alternatives after the intervention. However, for the question about dental biofilm, no significant improvement was observed (*p* > 0.05) (Table 1).

Significant differences were also observed between the pre‐test and the three‐month post‐test in responses about knowledge of dental caries, periodontal disease, oral hygiene practices, and preventive dentistry, indicating retention of learning. On the other hand, no significant changes were found regarding the definition of dental caries and dental biofilm (*p* > 0.05). In addition, responses remained consistent across all three questionnaires for questions about foods related to caries onset, the eruption age of the first permanent tooth, the ideal age for a child's first dental visit, fluoride function, and prior oral health activities conducted with students (Table 1).

These results should be interpreted with caution due to the small sample size, predominance of female participants, and the relatively short three‐month follow‐up period.

## 4 Discussion

The present study evaluated the oral health knowledge of elementary school teachers, comparing their responses before the educational intervention (pre‐test), immediately after (immediate post‐test), and three months later (post‐test). The results showed that teachers had insufficient knowledge in the pre‐test, but the intervention led to improvements in most questions, both immediately and three months after the intervention. The novelty of this study lies in its specific focus on the training of Brazilian teachers, an aspect that is still little explored in the literature. This underscores the importance of ongoing training programs aligned with public health policies.

The training program resulted in a clear and significant increase in teachers' knowledge, as evidenced by their correct answers after training. These findings align with previous studies showing that educational health programs positively impact knowledge and practices [1, 8, 9, 11, 12, 13, 24, 25, 26]. However, they differ from a study conducted in Indonesia, which reported no significant changes in knowledge, attitudes, or practices following a similar intervention [27].

Knowledge retention is a critical component in assessing the effectiveness of educational programs. The results of this study indicate that teachers retained part of the information learned, particularly regarding oral hygiene and preventive dentistry, even after three months. According to previous literature [15], knowledge retention is often used as an indicator of success in educational programs, being essential to ensure the practical application of information. Furthermore, repeating and reinforcing content regularly is essential for consolidating long‐term learning [27]. Single‐session interventions, like the one used in this study, offer a strategic approach in public health, designed to optimize time and resources while still delivering meaningful outcomes [28].

Despite the advances observed in many areas, the educational intervention did not significantly enhance teachers' understanding of cariogenic foods and the concept of dental biofilm. This difficulty may be related to the complexity of the concepts or how they were addressed during the intervention. Studies suggest that complex health topics, such as the composition of dental biofilm, require more interactive and personalized teaching methods to ensure effective comprehension and application. These approaches may include practical activities, simulations, or the use of audiovisual resources, which help reinforce learning and improve understanding [10, 11, 29, 30].

Furthermore, for questions related to cariogenic foods, the eruption age of the first permanent tooth, the ideal age for a child's first dental visit, fluoride function, and previous experience in teaching oral health to students, teachers maintained consistent answers across all three questionnaires. This suggests that some core concepts were already part of their existing knowledge and remained stable over time. Studies conducted in Haiti and Iran support this observation, showing that while educational interventions can improve certain behaviors and oral health practices, teachers' basic knowledge on fundamental topics often remains unchanged, even with the introduction of new information [13, 31].

The findings indicate that, although the intervention effectively promoted initial knowledge acquisition, some areas showed limited knowledge retention over time. This highlights the need for more frequent and sustained educational interventions. The literature supports this observation, emphasizing that repetition and reinforcement are essential to ensure that knowledge is retained and applied in practice [32]. Continuous reinforcement in oral health education programs can be fundamental for knowledge retention [30]. Systematic reviews recommend that educational programs should include periodic review and update components to promote lasting behavioral changes and improve oral health outcomes [9, 29].

The significant improvement in teachers' knowledge of oral health has direct implications for student education. Well‐informed teachers are better equipped to incorporate oral health content into their lessons, thereby encouraging healthier behaviors among students. Previous studies show that training programs focused on teachers enhance not only their knowledge and attitudes but also their ability to educate and motivate students toward proper oral hygiene [19, 22, 25]. In this sense, elementary schools, through teachers, play a crucial role in influencing children's health behaviors, especially during their formative years [9, 33]. Therefore, this study reinforces the importance of continuous training programs for educators, as such initiatives can improve both oral and general health outcomes in schoolchildren.

The present study has some limitations that must be considered when interpreting the results. Firstly, the final sample size consisted of 53 teachers who completed all questionnaires, resulting in a 36% loss. Despite the losses, this is a common limitation in intervention studies [34]. Additionally, the sample consisted exclusively of women, which reduces gender diversity [18]. However, this reflects the actual demographic profile of educators in Veranópolis, where the study was conducted. Another limitation is the use of self‐reported questionnaires, which may be subject to response bias. To mitigate this, the researcher remained available to assist participants and clarify doubts throughout the study. Finally, the follow‐up duration of three months after the intervention also raises questions about long‐term knowledge retention.

Future studies should investigate longer and more diverse educational interventions that reinforce both immediate learning and long‐term retention. Extending the follow‐up period beyond three months is essential to maintain and apply knowledge in classroom settings over time, as recommended by prior research [26]. Increasing sample size, improving gender representation, and including participants from larger municipalities could also enhance the generalizability of results.

Finally, this study contributes not only to improving oral health in the school environment but also demonstrates that teacher training through structured educational interventions can significantly improve their knowledge. These effects are maintained for at least three months, emphasizing the importance of incorporating regular oral health training into school health policies. Empowering teachers as agents of health promotion has the potential to foster lifelong healthy habits in students and build a culture of prevention from an early age.

## 5 Conclusions

This study concludes that the educational intervention improved elementary school teachers' knowledge of oral health, including concepts related to dental caries, periodontal disease, oral hygiene practices, and preventive dentistry. The immediate improvements and those observed three months after the intervention indicate that educational strategies are effective in raising awareness of oral health among teachers, who can serve as important mediators of oral health knowledge for their students.

## Ethics Statement

The study was approved by the Research Ethics Committee of ATITUS Educação (CAAE number 6691366). The teachers participated in the research after signing a free informed consent form. Furthermore, all schools and education departments agreed to participate in this research.

## Conflicts of Interest

The authors declare no conflicts of interest.

## Data Availability

The data that support the findings of this study are available from the corresponding author upon reasonable request.
